# CRISPR/Cas9-based genome editing in mice uncovers 13 testis- or epididymis-enriched genes individually dispensable for male reproduction^†^

**DOI:** 10.1093/biolre/ioaa083

**Published:** 2020-05-26

**Authors:** Jiang Sun, Yonggang Lu, Kaori Nozawa, Zoulan Xu, Akane Morohoshi, Julio M Castaneda, Taichi Noda, Haruhiko Miyata, Ferheen Abbasi, Hossam H Shawki, Satoru Takahashi, Darius J Devlin, Zhifeng Yu, Ryan M Matzuk, Thomas X Garcia, Martin M Matzuk, Masahito Ikawa

**Affiliations:** 1 Department of Experimental Genome Research, Research Institute for Microbial Diseases, Osaka University, Suita, Osaka, Japan; 2 Graduate School of Medicine, Osaka University, Suita, Osaka, Japan; 3 Center for Drug Discovery, Baylor College of Medicine, Houston, Texas, USA; 4 Department of Pathology and Immunology, Baylor College of Medicine, Houston, Texas, USA; 5 Graduate School of Pharmaceutical Sciences, Osaka University, Suita, Osaka, Japan; 6 Department of Comparative and Experimental Medicine, Graduate School of Medical Sciences, Nagoya City University, Nagoya, Japan; 7 Department of Anatomy and Embryology, Faculty of Medicine, University of Tsukuba, Tsukuba, Ibaraki, Japan; 8 Department of Biology and Biotechnology, University of Houston-Clear Lake, Houston, Texas, USA; 9 The Institute of Medical Science, The University of Tokyo, Tokyo, Japan

**Keywords:** male contraceptive, CRISPR/Cas9, knockout mice, male infertility, testis, epididymis

## Abstract

Developing a safe and effective male contraceptive remains a challenge in the field of medical science. Molecules that selectively target the male reproductive tract and whose targets are indispensable for male reproductive function serve among the best candidates for a novel non-hormonal male contraceptive method. To determine the function of these genes in vivo*,* mutant mice carrying disrupted testis- or epididymis-enriched genes were generated by zygote microinjection or electroporation of the CRISPR/Cas9 components. Male fecundity was determined by consecutively pairing knockout males with wild-type females and comparing the fecundity of wild-type controls. Phenotypic analyses of testis appearance and weight, testis and epididymis histology, and sperm movement were further carried out to examine any potential spermatogenic or sperm maturation defect in mutant males. In this study, we uncovered 13 testis- or epididymis-enriched evolutionarily conserved genes that are individually dispensable for male fertility in mice. Owing to their dispensable nature, it is not feasible to use these targets for the development of a male contraceptive.

## Teaser

Thirteen testis- or epididymis-enriched genes are individually dispensable for male fertility based on phenotypic analyses of mutant mice produced by the CRISPR/Cas9 system.

## Introduction

Contraceptive drugs have been available only for women for the past several decades, during which the world population underwent approximately a 2.4-fold increase, from 3 billion in 1960 to over 7.2 billion in 2015 [1]. The Strategic Plan 2000 of the National Institute of Child Health and Human Development states that uncontrolled fertility is one of the most pressing public health issues we face worldwide [2]. In the United States, approximately 49% of women have experienced an unintended pregnancy [3]. With such a high rate of unplanned pregnancies, there is an increased risk of adverse infant health outcomes, elevated rates of maternal mortality [4], ethical issues, social problems, and increased healthcare expenditure [5].

A lack of alternatives for contraception in men is partially responsible for these problems. However, the development of a safe and effective male contraceptive approach remains a long-term challenge in the field of medical science. So far, testosterone analogs have been the only approach in clinical trials that alter the production of endogenous androgens to produce a contraceptive effect in men [6]. To overcome the deficiency of contraceptive alternatives for men, non-hormonal male contraceptive pills can be developed to target the male reproductive tract in a reversible manner without jeopardizing normal hormone levels in men [7].

Due to extreme similarities between human and mouse genomes, the mouse has been serving as a powerful model organism for studying human diseases for decades [8]. Previous studies have demonstrated that more than 2300 genes are predominantly expressed in the male germ line, hundreds of which may be essential for male reproductive functions [9]. Nevertheless, only a limited number of testis- and epididymis-enriched genes have been documented regarding their roles in underpinning normal spermatogenesis and sperm functions such as glycosylphosphatidylinositol-anchored protein TEX101 [10], A kinase anchor protein AKAP4 [11], and sperm-specific glycolytic enzyme glyceraldehyde 3-phosphate dehydrogenase-S [12], receptor tyrosine kinase, ROS proto-oncogene ROS1 [13], and epididymal receptor HE6 [14]. To further assess their potential as male contraceptive targets, the protein functions and interactions, spatiotemporal expression in testis and epididymis, and domain structures are yet to be elucidated [15, 16].

Utilizing the CRISPR/Cas9 genome editing system, generation of knockout mice has become an effective and efficient way to interrogate the functional requirement of many genes in vivo [17–21]*.* In this study, we generated 11 knockout mouse lines bearing large deletions in the target loci (i.e., *4921507P07Rik*, *Allc*, *Fam229b*, *Fscb*, *Iqca*, *Lelp1*, *Spata24*, *Hdgfl1*, *Tmem97*, *Lrcol1*, and *Tmem114*) and two mutant mouse lines carrying indel mutations (i.e., *Cabs1* and *Eddm3b*). Among these 13 genes of interest, *C7orf31* (ortholog of *4921507P07Rik* in human), *ALLC*, *CABS1*, *FAM229B*, *FSCB*, *IQCA*, *LELP1*, *SPATA24*, *HDGFL1*, and *TMEM97* show testis-enriched expression in human, whereas *EDDM3B*, *LRCOL1*, and *TMEM114* were predominantly expressed in human epididymis. Phenotypic analyses revealed that all of these 13 genes are individually dispensable for male fertility in mice. Such reproductive tract-enriched proteins cannot serve as targets for male contraceptive development because of their dispensable nature.

## Materials and methods

### Animals

Wild-type B6D2F1 and ICR mice used for experiments in the laboratory of MI were purchased from Japan SLC, Inc. or CLEA Japan, Inc. for knockout mice production and phenotypic analyses. Wild-type mice obtained by intercrosses between C57BL6 and 129S5/SvEvBrd mice were used in the laboratories of MMM and TXG for analyzing the patterns of tissue expression for all candidate genes. All animal experiments were approved by the Animal Care and Use Committee of the Research Institute for Microbial Diseases, Osaka University and the Institutional Animal Care and Use Committee of Baylor College of Medicine.

### Phylogenetic analyses

The phylogenetic trees of candidate genes were constructed by GENETYX software (GENETYX Corp., Tokyo, Japan) using the neighbor-joining method based on their amino acid sequences.

### Digital PCR

Digital polymerase chain reaction (PCR) was conducted as previously described to depict the tissue expression of each candidate gene [19]. Sequences of various tissues were downloaded from Sequence Read Archive and aligned against the human genome (GRCh38) or mouse genome (GRCm38) by HISAT2 after being trimmed using TrimGalore. After quantification using featureCounts, the gene expression in each tissue was batch corrected by RUVR to remove the unwanted variation. Differential gene expression was further determined for each non-reproductive tissue against each reproductive tissue by EdgeR. The expression data for reproductive tissues were retrieved from 18 purified human germ cell data sets [22], 5 human testis data sets [23], 6 human epididymis segment data sets [24], whereas data for the 26 non-reproductive human tissues and the 14 non-reproductive mouse tissues were obtained from 118 and 62 additional data sets, respectively [25, 26].

### In silico analyses of mRNA expression in spermatogenic cells

Messenger RNA (mRNA) expression of the genes of interest in testicular germ and somatic cells was examined in silico by 10x Genomics Loupe Browser using single-cell RNA-sequencing (scRNA-seq) data set published previously [27].

### Mutant mouse production by CRISPR/Cas9

All mutant mice were generated by the CRISPR/Cas9 genome editing system. Single-guide RNAs (sgRNAs) were designed and off-target analyses were performed using the online software CRISPRdirect (crispr.dbcls.jp) [28]. The editing efficiency of each sgRNA was evaluated by the intensity of the fluorescence signal obtained from co-transfecting HEK293T cells with a pX459 plasmid (Addgene #62988) bearing the sgRNA sequence and a pCAG–EGxxFP plasmid (Addgene #50716) carrying the target sequence as described previously [29].

Wild-type fertilized eggs were collected from superovulated B6D2F1 female mice that had been paired with B6D2F1 males. To generate knockout mice via zygote electroporation, CRISPR RNA (crRNA)/trans-activating crRNA (tracrRNA)/Cas9 ribonucleoprotein complexes were introduced into two-pronuclear eggs using a NEPA21 super electroporator (NEPA GENE, Chiba, Japan) [30]. To generate mutant mice via zygote microinjection, pX459 plasmids encoding the sgRNAs and Cas9 protein were microinjected into the pronuclei of zygotes [29]. The treated zygotes were then cultured in potassium simplex optimization medium (KSOM) [31] to two-cell stage and transplanted into the oviducts of 0.5-day pseudopregnant ICR females. The founder generation was obtained by natural delivery or Cesarean section and genotyped by PCR and the mutant alleles were subsequently verified by Sanger sequencing.

The mutant mouse lines generated by zygote electroporation or microinjection are indicated in Supplementary Table S1. The primers and PCR conditions used for genotyping are enumerated in Supplementary Table S2.

### Fertility tests for mutant males

Upon sexual maturity, homozygous or compound heterozygous mutant males were caged with three 8-week-old wild-type B6D2F1 females for at least 8 weeks. Exceptionally, *Cabs1* homozygous mutant males were individually paired with two wild-type females for 8 weeks; *Eddm3b* and *Lrcol1* mutant males were individually caged with one 8-week-old wild-type (in-house hybrid, C57BL/6J×129S5/SvEvBrd) female for 16 weeks. For each mutant line, at least two males were tested for a valid statistical interpretation. Three B6D2F1 wild-type males were tested in parallel as positive controls. The number of pups was recorded at birth. The average litter size was calculated by dividing the total number of pups with the number of litters.

### Analyses of testis weights and sperm motility

After the fertility tests, homozygous or compound heterozygous mutant male mice were anesthetized and euthanized by cervical dislocation. Testis weight relative to body weight was compared between wild-type and mutant males. The motility of cauda epididymal spermatozoa was analyzed using Hamilton Thorne CEROS II system (Hamilton Thorne Biosciences, Beverly, MA) at 10 min and 2 h of incubation in Toyoda, Yokoyama, Hoshi (TYH) medium.

### Analyses of testis and epididymis histology and sperm morphology

Testes and epididymides were fixed in Bouin solution and embedded in paraffin wax. Paraffin sections were stained with periodic acid (Nacalai Tesque, Kyoto, Japan) and Schiff reagent (Wako, Osaka, Japan) and counterstained with Mayer hematoxylin solution (Wako, Osaka, Japan). The caudal epididymal spermatozoa were dispersed in TYH medium and observed under an Olympus BX53 phase contrast microscopy.

### Statistical analysis

Statistical analyses were carried out using the Student *t*-test. Differences were recognized as statistically significant when the *P* value was lower than 0.05.

## Results

### In silico expression and conservation analyses of candidate genes

**Figure 1:**
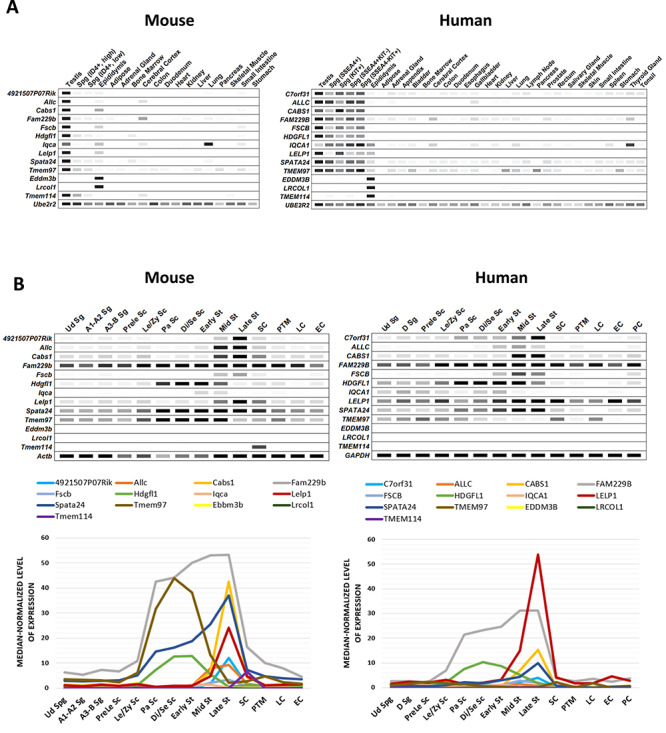
In silico analyses of the expression patterns of specific genes in multiple tissues and spermatogenic cells. (**A**) Digital PCR indicates the expression patterns of several genes of interest in mouse and human tissues. The average transcript per million (TPM) value per tissue per gene was generated from 77 published mouse RNA-seq data sets and 147 human RNA-seq data sets, respectively. All 13 genes showed predominant or restricted expression in both mouse and human testes or epididymides. Black = maximum value TPM. (**B**) ScRNA-seq data depicting the median-normalized levels of mRNA expression of the 13 genes of interest during human and mouse spermatogenesis. **Upper panel**: The level of mRNA expression in each cell type is indicated by the band intensity. The threshold of median-normalized reads in mouse is set to nine, whereas the threshold in human is set to four. **Lower panel**: Linear graphs show the mRNA expression of each gene at various testicular germ and somatic cells. Ud Sg, undifferentiated spermatogonia; A1–A2 Sg, A1–A2 differentiating spermatogonia; A3–B Sg, A3–A4–In–B differentiating spermatogonia; D Sg, differentiated spermatogonia; Prele Sc, preleptotene spermatocytes; Le/Zy Sc, leptotene/zygotene spermatocytes; Pa Sc, pachytene spermatocytes; Di/Se Sc, diplotene/secondary spermatocytes; Early St, early round spermatids; Mid St, mid round spermatids; Late St, late round spermatids; SC, Sertoli cells; PTM, peritubular myoid cells; LC, Leydig cells; EC, endothelial cells; PC, perivascular cells.

Two major criteria for a human male contraceptive target are (i) male reproductive tract specificity to minimize potential side effects and (ii) the presence of a mouse ortholog for functional validation in an animal model. As disparities were found among different published databases, we further confirmed the expression patterns of the 13 genes of interest by digital PCR. As shown in Figure 1A, all genes exhibited predominant expression in the testes or epididymides of humans and mice. Using published scRNA-seq data generated from mouse and human spermatogenic cells at different stages, testis-enriched genes, *4921507P07Rik*, *Allc*, *Cabs1*, *Fam229b*, *Fscb*, *Iq*, *Lelp1*, *Spata24*, *Hdgfl1*, and *Tmem97*, were found to exhibit elevated mRNA expression in late spermatocytes or early spermatids in both mouse and human, whereas the mRNA expression of epididymis-enriched genes *Eddm3b* and *Lrcol1* was below the level of detection in the male germ line of both species (Figure 1B). Notably, *Tmem114*, which showed a discrepancy of tissue expression bias in mouse and human (Figure 1A), was found to be expressed in the Sertoli cells in mouse but not expressed in the testicular germ or somatic cells in human (Figure 1B).

**Figure 2:**
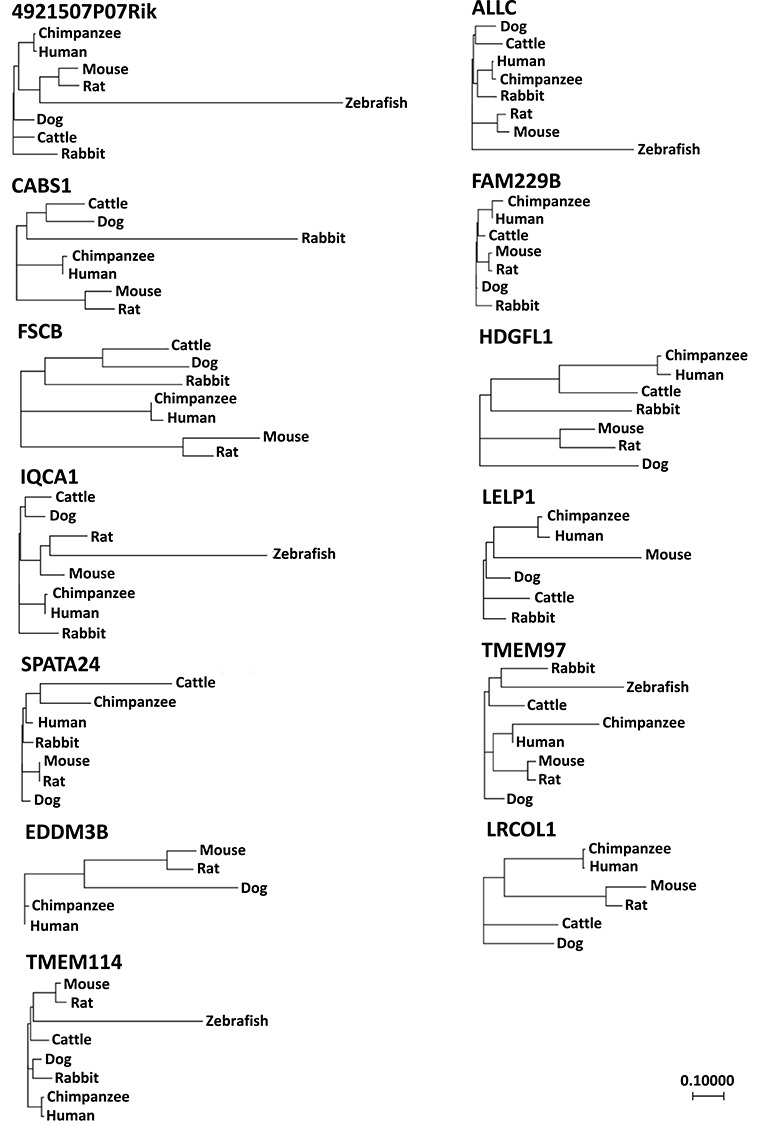
Phylogenetic analysis of the 13 testis- or epididymis-enriched genes.

Phylogenetic analyses indicated that all genes are conserved among mammals. *4921507P07Rik*, *Allc*, *Iqca*, *Tmem97*, and *Tmem114* were also found in zebrafish (Figure 2).

**Figure 3:**
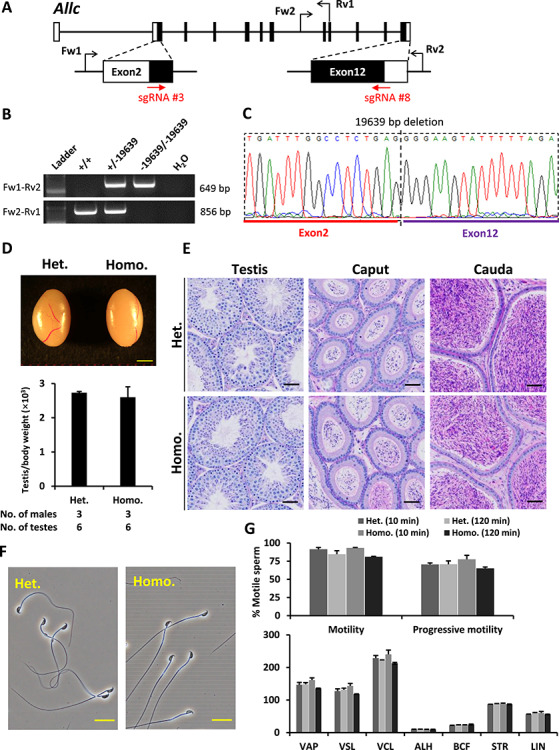
Phenotypic analysis of *Allc* knockout male mice. (**A**) Genomic structure and knockout strategy of *Allc*. Two sgRNAs were designed to target the first coding exon (Exon 2) and the last coding exon (Exon 12). Four primers (Fw1, Fw2, Rv1, and Rv2) were designed for genotyping the mutant mice. (**B**) Mutant and wild-type alleles were detected by genomic PCR using primer sets Fw1–Rv2 and Fw2–Rv1, respectively. (**C**) DNA sequence of the knockout allele was determined by Sanger sequencing. (**D**) Testis appearance and testis to body weight ratios of *Allc* heterozygous and homozygous knockout mice. Scale bar = 2 mm. (**E**) Histological analyses of testes and epididymides in *Allc* heterozygous and homozygous knockout mice. Scale bars = 50 μm. (**F**) Morphology of cauda epididymal spermatozoa in *Allc* heterozygous and homozygous knockout mice. Scale bars = 20 μm. (**G**) Analysis of sperm motility in *Allc* heterozygous and homozygous knockout mice. Sperm motility and kinetic parameters were measured at 10 and 120 min of incubation in TYH media. VAP, average path velocity; VSL, straight line velocity; VCL, curvilinear velocity; ALH, amplitude of lateral head displacement; BCF, beat cross frequency; STR, straightness; LIN, linearity.

### Phenotypic analyses of mutant males

To investigate if the candidate genes are essential for male reproduction, we disrupted the genes individually by the CRISPR/Cas9-mediated genome editing system. The mutant alleles were detected by genomic PCR and confirmed by Sanger sequencing. The mutated loci of all mouse lines were summarized in Supplementary Table S3. The Sanger sequencing result of *4921507P07Rik* wild-type and homozygous knockout mice is shown as an example in Supplementary Figure S1. Herein, phenotypic analyses of *Allc*, *Fscb*, and *4921507P07Rik* knockout mice are shown as examples for all genes.

*Allc* was deleted completely by introducing crRNA/tracrRNA/Cas9 complexes into two-pronuclear eggs via electroporation. The two sgRNAs targeted the first coding exon (Exon 2) and the last coding exon (Exon 12; Figure 3A) of *Allc*. Off-target effect and cleavage efficiency of the two sgRNAs was evaluated using the CRISPRdirect software and EGxxFP reporter assay, respectively. Mutant allele carrying a 19 639-bp deletion was identified by genomic PCR and Sanger sequencing (Figure 3B and C) using the four primers presented in Figure 3A and Supplementary Table S2. No statistically significant difference was observed in the testis sizes and weights between the homozygous and heterozygous knockout mice (Figure 3D). Same as in the control samples, histological sections of knockout testes and epididymides showed normal spermatogenesis in the seminiferous tubules and normal sperm morphology and density in the caput and cauda epididymides (Figure 3E and Supplementary Figure S2). Moreover, the depletion of *Allc* did not affect the morphology of mature spermatozoa (Figure 3F). Computer-assisted sperm analysis revealed that *Allc-*null spermatozoa exhibited normal motility compared with control spermatozoa (Figure 3G).

**Figure 4:**
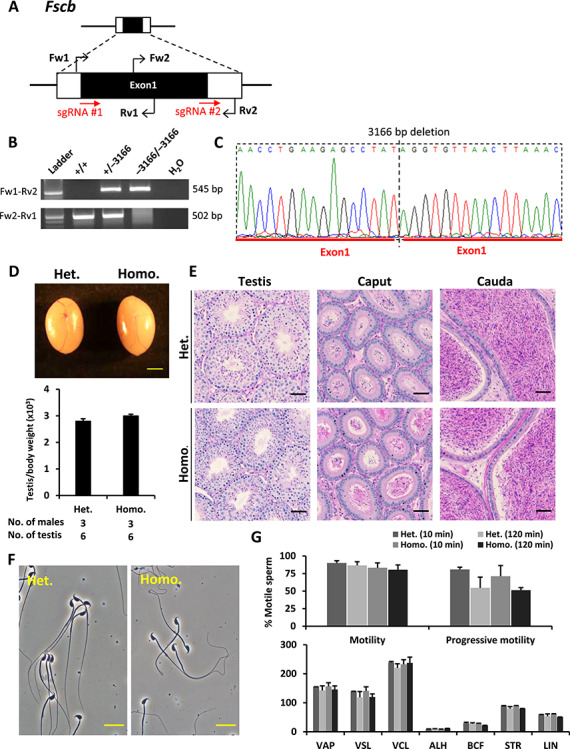
Phenotypic analysis of *Fscb* knockout male mice. (**A**) Genomic structure and knockout strategy of mouse *Fscb*. Two sgRNAs were designed to target the 5′ and the 3′ region, respectively, of the coding exon (Exon 1). Four primers (Fw1, Fw2, Rv1, and Rv2) were designed for genotyping. (**B**) Mutant and wild-type alleles were detected by genomic PCR using primer sets Fw1–Rv2 and Fw2–Rv1, respectively. (**C**) DNA sequence of the knockout allele was determined by Sanger sequencing. (**D**) Testis appearance and testis to body weight ratios of *Fscb* heterozygous and homozygous knockout mice. Scale bar = 2 mm. (**E**) Histological analyses of testes and epididymides in *Fscb* heterozygous and homozygous knockout mice. Scale bars = 50 μm. (**F**) Morphology of cauda epididymal spermatozoa in *Fscb* heterozygous and homozygous knockout mice. Scale bars = 20 μm. (**G**) Analysis of sperm motility in *Fscb* heterozygous and homozygous knockout mice. Sperm motility and kinetic parameters were measured at 10 and 120 min of incubation in TYH media.

*Fscb* is a gene containing a single coding exon. To disrupt this gene, two sgRNAs as indicated in Figure 4A were designed to target the 5′and 3′ region, respectively, of the coding exon (Exon 1). Primers for identifying the mutant allele are presented in Figure 4A and B and Supplementary Table S2. Compound heterozygotes carrying a 3166 or 3164-bp deletion in each allele or homozygotes carrying a 3166-bp deletion in both alleles (Figure 4C) were used for analyzing the knockout phenotype. *Fscb* knockout mice exhibited normal testicular size and weight (Figure 4D), normal testicular and epididymal histology and spermatogenesis (Figure 4E and Supplementary Figure S3), and normal sperm morphology and motility (Figure 4F and G).

**Figure 5:**
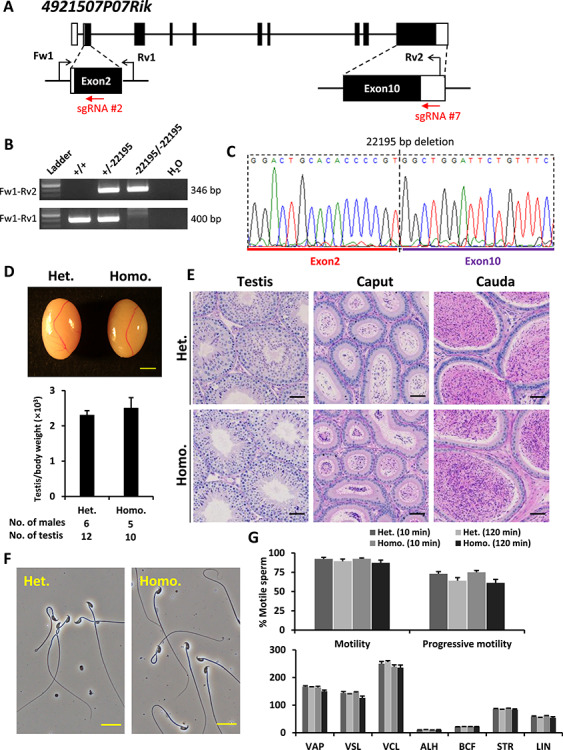
Phenotypic analysis of *4921507P07Rik* knockout male mice. (**A**) Genomic structure and knockout strategy of mouse *4921507P07Rik*. Two sgRNAs were designed to target the first coding exon (Exon 2) and the 3′ UTR region of the last coding exon (Exon 10). Three primers (Fw1, Rv1, and Rv2) were designed for genotyping. (**B**) Mutant and wild-type alleles were detected by genomic PCR using primer sets Fw1–Rv2 and Fw1–Rv1, respectively. (**C**) DNA sequence of the knockout allele was determined by Sanger sequencing. (**D**) Testis appearance and testis to body weight ratios of *4921507P07Rik* heterozygous and homozygous knockout mice. Scale bar = 2 mm. (**E**) Histological analyses of testes and epididymides in *4921507P07Rik* heterozygous and homozygous knockout mice. Scale bars = 50 μm. (**F**) Morphology of cauda epididymal spermatozoa in *4921507P07Rik* heterozygous and homozygous knockout mice. Scale bars = 20 μm. (**G**) Analysis of sperm motility in *4921507P07Rik* heterozygous and homozygous knockout mice. Sperm motility and kinetic parameters were measured at 10 and 120 min of incubation in TYH media.

Mutant mice carrying a 22 195-bp deletion in the locus of *4921507P07Rik* were generated using two sgRNAs targeting Exons 2 and 10, respectively. Primers used to detect the mutant alleles are presented in Figure 5A and Supplementary Table S2. The testis size, weight and histology, spermatogenesis, and sperm morphology and motility were normal in homozygous knockout males (Figure 5 and Supplementary Figure S4).

Analyses of testis weights, testis and epididymis histology and sperm morphology in other mutant mouse lines were presented in Supplementary Table S4 and Supplementary Figures S5 and S6.

### Fertility tests for mutant male mice

To examine the fecundity of knockout males, individual knockout male mice were caged with up to three wild-type female mice for at least 2 months. The fecundity of three wild-type males was tested in parallel as positive controls. The average litter size of each knockout male was approximately eight pups per litter, comparable to the wild-type controls as shown in Table 1. Thus, all of the mutant males in this study showed normal fertility.

**Table 1:** Outcomes of the fertility tests for the 13 mutant mouse lines. Compound heterozygotes carrying different mutations in each allele (−3166/−3164) were used for testing the fertility of *Fscb* knockout males. Exceptionally, *Cabs1* mutant males were individually paired with two wild-type females and *Eddm3b* and *Lrcol1* mutant males were individually caged with one wild-type female.

Gene symbol	Genotype	No. of males	No. of pups	No. of litters	Mating period	Average litter size
Wild-type	+/+	3	264	30	10 weeks	8.8 ± 0.6
*4921507P07Rik*	−22195/−22195	3	185	19	8 weeks	9.7 ± 1.2
*Allc*	−19639/−19639	3	189	24	8 weeks	9.1 ± 1.4
*Cabs1*	−5/−5	3	121	13	8 weeks	9.3 ± 2.6
*Fam229b*	−3266 + 17/−3266 + 17	3	233	23	8 weeks	10.1 ± 1.7
*Fscb*	−3166/−3164	3	192	20	8 weeks	9.6 ± 0.9
*Hdgfl1*	−1095/−1095	3	231	25	12 weeks	9.2 ± 2.3
*Iqca*	−107633/−107633	2	139	16	8 weeks	8.7 ± 2.1
*Lelp1*	−577 + 118/−577 + 118	2	138	14	8 weeks	9.9 ± 1.9
*Spata24*	−5638/−5638	3	223	23	9 weeks	9.7 ± 0.7
*Tmem97*	−8286/−8286	3	255	26	12 weeks	8.5 ± 1.0
*Eddm3b*	−5/−5	4	133	15	16 weeks	9.0 ± 0.8
*Lrcol1*	−1196/−1196	5	187	20	16 weeks	9.5 ± 1.4
*Tmem114*	−15261/−15261	3	251	28	12 weeks	9.0 ± 2.6

## Discussion

Contraceptives for females have been available for preventing unintended pregnancy for quite a long time, but many women do not use them because of potential side effects and inconvenience. In developing countries, more than 700 000 maternal deaths are related to unplanned pregnancies from 1995 to 2000 [6]. Developed countries also show high rates of unintended pregnancy—more than 50% women have experienced an unplanned pregnancy [32]. The lack of contraceptives for men is partially responsible for the high occurrence of unplanned pregnancies and the associated maternal mortality. At present, male contraceptives mainly consist of condoms and vasectomy. These methods are not ideal, because contraceptive failure may occasionally occur using condoms and vasectomy may neither assure successful reversal nor it is convenient. The only male contraceptive drugs in clinical trials are based on hormonal disruption [6], where the endogenous androgen production is inhibited, thereby impairing sperm production [33]. Although hormonal contraceptives appear to be safe, additional studies are required to investigate the long-term effects of testosterone administration for overall male health, because contraceptives would be used by healthy individuals for extended periods of time. Therefore, the development of non-hormonal male contraceptives is of immense importance.

When developing contraceptive drugs, one of the most important factors to be considered is target validation, which can be assessed by using knockout mice, antibodies, and known inhibitors. All of the 13 genes in this study have human orthologs and their amino acid sequences show high homology between mouse and human (Supplementary Figure S7**)**, suggesting conserved gene function across species. An expression bias toward the male reproductive organ is another important criterion for a candidate contraceptive target. In this study, all candidate genes showed elevated expression in the testes or epididymides of human and mouse as shown in Figure 1A. Although some genes exhibited minor expression in other tissues, we did not observe any abnormal appearance or behavior in the mutant mice.

As presented in Supplementary Figures S8 and S9, indel mutations disrupted the functions of *Cabs1* and *Eddm3b* by introducing premature stop codons. However, it is possible that the normal fecundity of the mutant males is attributed to the presence of functional truncated proteins produced by alternative splicing. To avoid such concerns, in the present study, all other 11 knockout mouse lines were generated by removing the entire open reading frames. To further enhance the screening stringency, all of the mutant mice were generated on a mixed background of B6D2F1/J mice. We deposited all of these mutant mouse lines to the bioresource center, which may be of value to other researchers for their studies **(**Supplementary Table S5**)**.

By using the Mouse Genome Informatics database (informatics.jax.org), UniProt (uniprot.org), NCBI’s Protein database (ncbi.nlm.nih.gov/protein), the web-based Simple Modular Architecture Research Tool (smart.embl-heidelberg.de), TMHMM2.0 (cbs.dtu.dk/services/TMHMM) [34], and SignalP5.0 (cbs.dtu.dk/services/SignalP) [35], the features of the proteins of interest, such as conserved domains, interacting proteins, subcellular localization, and the presence of a signal peptide and transmembrane domains, were gathered for predicting gene functions and their potential involvement in reproduction (Supplementary Table S6).

*Allc* contains two allantoicase domains, which are involved in purine degradation that facilitates the utilization of purines as secondary nitrogen sources under nitrogen-limited conditions. Although allantoicase activity is not detected in mammals [36, 37], the domain may have an alternative function in reproduction or the non-conserved region determines the function of ALLC. This protein could have been a druggable target for male contraceptive development, if *Allc* knockout males were sterile and enzymatic activity could be confirmed. However, our functional analysis utilizing the knockout strategy suggests that *Allc* is not a suitable target because of its dispensable nature.

CABS1, calcium binding protein, spermatid specific 1, is a novel calcium-binding protein predominantly expressed in the testis, specifically in steps 10–16 elongating spermatids [38]. It has been reported that the human ortholog of *Cabs1* is located to the gene cluster of the secretory calcium-binding phosphoproteins, indicating this molecule may have a crucial role in late spermiogenesis [39]. Fibrous sheath calcium-binding tyrosine-phosphorylation-regulated (CABYR)-binding protein, FSCB, has been characterized as a sperm protein that is a direct target of protein kinase A (PKA)-mediated tyrosine phosphorylation during sperm capacitation [40, 41]. CABYR, which localized to the fibrous sheath of the flagellar principal piece, is indispensable for reproduction [42, 43]. It is well known that the cyclic adenosine monophosphate (cAMP)-dependent PKA signaling pathway plays critical roles in sperm motility, capacitation, and the acrosome reaction [44], and that PKA-dependent phosphorylation of FSCB is believed to activate spermatozoa motility by inhibited SUMOylation of two crucial proteins that are associated with PKA/A kinase activity [45]. These studies suggested that FSCB may be essential for male reproduction. However, *Fscb* knockout males were found to have normal fecundity, suggesting that the depletion of *Fscb* does not affect sperm motility, capacitation, or the acrosome reaction.

In recent years, many of the testis-enriched genes essential for male reproduction were found by using the knockout approach [46]. Some of them, such as bromodomain testis-specific protein (BRDT) and protein phosphatase 3 catalytic subunit gamma (PPP3CC), were recognized as candidate targets for non-hormonal male contraceptives.

BRDT is a testis-specific bromodomain motif-containing protein [47, 48]. *Brdt* knockout males are infertile due to abnormal spermatogenesis [49, 50]. The inhibition of BRDT using a small-molecule bromodomain inhibitor JQ1 achieves a complete and reversible contraceptive effect without influencing the male mouse testosterone levels and mating behaviors, or the well-being of their offspring [51, 52].

Calcineurin is a Ca^2+^- and calmodulin-dependent serine–threonine phosphatase that is crucial for calcium signaling [53, 54]. PPP3CC has been identified as a catalytic subunit isoform of calcineurin in mammals [55]. *Ppp3cc* knockout males showed normal spermatogenesis and sperm counts and morphology, yet displayed infertility due to defective zona pellucida penetration [56]. Male mice exhibited defective sperm morphology and motility within 4 to 5 days after treatment of calcineurin inhibitors, such as cyclosporine A and FK506. The mice recovered from sterility after one week of stopping treatment [57, 58], indicating that sperm calcineurin is a potential target for the development of reversible and rapid non-hormonal male contraceptives [1, 56].

In conclusion, we disrupted 13 testis- or epididymis-enriched genes in mice by the CRISPR/Cas9 system. Fertility tests and phenotypic analyses of the mutant males revealed that all of these genes are dispensable for male fecundity in mice and are thus not suitable for further development as a male contraceptive target. Using this in vivo approach, essential genes that have novel functions in male reproduction can be efficiently identified. Our progress toward not only understanding the key molecules underpinning normal male reproduction, but also the identification of druggable targets for non-hormonal male contraceptives, has been vastly accelerated.

## Supplementary Material

SI_R1_ioaa083
