# Supplementary material for: CRISPR/Cas9-based genome editing in mice uncovers 13 testis- or epididymis-enriched genes individually dispensable for male reproduction
Source: Biol Reprod. 2020 May 26;103(2):183–94. doi: 10.1093/biolre/ioaa083 (PMC7401351; doi:10.1093/biolre/ioaa083)
Supplement: SI_R1_ioaa083 [file si_r1_ioaa083.pdf]

Supplementary Table S1. Single-guide RNAs targeting the genes' upstream (U) and downstream (D) regions used for generating knockout mice. Efficiency of embryo transplantation was presented using the number of total pups delivered by pseudopregnant mice divided by the number of total embryos used for oviduct transplantation (Total pups/embryos transplanted). Efficiency of genome editing was determined by the number of pups carrying enzymatic mutations divided by the number of pups subjected to genotyping (GM pups/pups genotyped).

Gene symbol	KO strategy	Total pups/embryos transplanted	GM pups/ pups genotyped	PAM	sgRNA sequences	
4921507P07Rik	Zygote electroporation	14/90 (15.6%)	3/14 (21.5%)	CGG	U	GGCCGAATAGTTGTTTCGTA
				AGG	D	AACAGAATCCAGCCAAATGC
Allc	Zygote electroporation	15/56 (27%)	1/15 (6%)	GGG	U	GATTTGGCCTCTGAGTGCGT
				AGG	D	TAGGGAAACCCTTGAGCCGA
Cabs1	Zygote injection	15/77 (19%)	5/11 (45%)	TGG	U	AGTCATTTACTGAAGTAACG
Fam229b	Zygote electroporation	16/75 (21%)	15/16 (94%)	AGG	U	CTTCTTGAGCCCGCTAGCTA
				GGG	D	ATCATTAAGTCTTCTGTCGA
Fscb	Zygote electroporation	17/100 (23%)	10/17 (59%)	AGG	U	CCTGAAGAGCCTATATCATT
				AGG	D	TAAAGAACTGAGCTATCGTC
Hdgfl1	Zygote electroporation	15/80 (18.8%)	9/15 (60%)	AGG	U	AGCAGCAGCGGGACCTCTCG
				GGG	D	GTTGAAGACTGGGCCCTAGT
Iqca	Zygote electroporation	20/80 (25%)	5/20 (25%)	AGG	U	GTTCCCCCAACTTCGGCAGT
				AGG	D	TCTTCCTCCTCTCGGTACAC
Lelp1	Zygote electroporation	6/33 (18%)	3/6 (50%)	AGG	U	TGAAAGCTTCTCGAGAAAAC
				AGG	D	CCATGCCTACCCCGAAAGTG
Spata24	Zygote electroporation	16/40 (40%)	13/16 (81%)	GGG	U	ACAGAGCACCCTGGCAACCG
				AGG	D	GACAGTGCCACTGCTCATGG
Tmem97	Zygote electroporation	4/46 (8.7%)	3/4 (75%)	TGG	U	CAGCTAGGCGCTGTGTCGAG
				TGG	D	GCAGTGCTGTGGTAGCGCTT
Eddm3b	Zygote electroporation	10/79 (13%)	1/10（10%）	GGG	U	TCAGGCCTTGGTCACCGTCC
Lrcol1	Zygote electroporation	6/81 (7.4%)	3/6 (50%)	TGG	U	GTGAGATGTCTGTCTCGGTG
				TGG	D	CACATTCCAAAAACACGGTC
Tmem114	Zygote electroporation	20/65 (30.8%)	11/20 (55%)	TGG	U	TGCGGGTGGGATCGCGTAAG
				TGG	D	CCAGGAGGCGCAGGGCCTTT

Supplementary Table S2. Primers and PCR conditions used for genotyping.

Gene symbol	Allele	Forward primer	Reverse primer	Band size	Annealing	Elongation
4921507P07Rik	WT	TAAGCCTCTGGTTTTTGTGC	TACTCATGCTTTGCCTTCTCC	400 bp	65°C, 30 s	72°C, 30 s
	KO	TAAGCCTCTGGTTTTTGTGC	GTCAGGACATTATTTTGGTCC	346 bp	65°C, 30 s	72°C, 30 s
Allc	WT	GCCTTGCTGTGTTGTAAGCC	ATGGACTTGGGTTCACCGAC	856 bp	65°C, 30 s	72°C, 1 min
	KO	CAGAGCTCTCACCGGTTCTC	AGCACCACATAGGGTATGTA	649 bp	65°C, 30 s	72°C, 45 s
Cabs1	WT	CTGTGAGAGTGCTCAGTGCC	CCCTTAGGTTTGGAGTTTGG	350 bp	65°C, 30 s	72°C, 30 s
	KO	CTGTGAGAGTGCTCAGTGCC	CCCTTAGGTTTGGAGTTTGG	345 bp	65°C, 30 s	72°C, 30 s
Fam229b	WT	AGGCAGGAAGCTATGTGAAGG	TACACAAGGGATAGGAAAGGTGA	422 bp	65°C, 30 s	72°C, 30 s
	KO	AGGCAGGAAGCTATGTGAAGG	GCTTAGCATGCAGAGCGCTC	347 bp	65°C, 30 s	72°C, 30 s
Fscb	WT	GGAACTGTAGAAGACCTGACTGC	GAGAAACTTCAGCAGAGCCC	502 bp	65°C, 30 s	72°C, 30 s
	KO	GTCCTAACAGAAACAAACCAAG	TAAGCAATCATTGTCCATGG	545 bp	65°C, 30 s	72°C, 30 s
Hdgfl1	WT	ACCAACATTTCTTAACATGG	ACAAAGTTGATGTCAACTGG	1514 bp	60°C, 30 s	60°C, 30 s
	KO	ACCAACATTTCTTAACATGG	ACAAAGTTGATGTCAACTGG	419 bp	60°C, 30 s	60°C, 30 s
Iqca	WT	GGGGTTGCTTGTGGAAACAC	TCAACAGCCTTCCTTCGAGT	461 bp	63°C, 30 s	72°C, 60 s
	KO	CGGCTCACATAAACAGGGCA	TCCATCAAGAACGGAGGTGAG	978 bp	63°C, 30 s	72°C, 60 s
Lelp1	WT	CCATGCCTACCCCGAAAGTG	CAGCCCCACAATGTTGTAGG	968 bp	65°C, 30 s	65°C, 30 s
	KO	CCATGCCTACCCCGAAAGTG	CAGCCCCACAATGTTGTAGG	509 bp	65°C, 30 s	65°C, 30 s
Spata24	WT	TCTGTTGCAGATGGTTCTCC	CAGGACTCTGCTCTTGACAC	542 bp	65°C, 30 s	72°C, 30 s
	KO	TTGGGGGATGAAATCCAGTC	AGCTCAGTAGAGGCGGGTGC	199 bp	55°C, 30 s	72°C, 30 s
Tmem97	WT	GTGACCATCTAGGCCAATGG	GGCAATATACTAACCTAAGG	591 bp	60°C, 30 s	72°C, 30 s
	KO	CTATAGCCCCGCTATATGGG	GGTGAGGTGCCAGAGTAGGG	552 bp	65°C, 30 s	72°C, 30 s
Eddm3b	WT	CAAAAGAGAGGGCATATAGGAGG	AGCAGAAGCAGGGCCCAGGC	296 bp	65°C, 30 s	72°C, 30 s
	KO	CAAAAGAGAGGGCATATAGGAGG	GAGCAGAAGCAGGGCCTTTT	292 bp	65°C, 30 s	72°C, 30 s
Lrcol1	WT	CAGCCAGGGCTACACGGAAA	GATGGGGCTGCATGACGATTGGG	491 bp	65°C, 30 s	72°C, 30 s
	KO	CAGCCAGGGCTACACGGAAA	GGTCTTTGATGAGCAGAACC	489 bp	65°C, 30 s	72°C, 30 s
Tmem114	WT	ACTCAGTCTGATCGTGATGG	TCCGCTGCTTCTACTTGTGG	567 bp	60°C, 30 s	60°C, 30 s
	KO	GGACGCTCGCCCCGATCCGG	AAAACTACCGGGTGACTAGG	587 bp	60°C, 30 s	60°C, 30 s

Supplementary Table S3. Sanger sequencing of mutant alleles in all mouse lines. Bases in uppercase indicate exon sequences and the ones in lowercase indicate intron sequences.

**Gene symbol**	**Upstream sequence** (25 bp)	**Mutation**	**Downstream sequence** (25 bp)
4921507P07Rik	TACTCTAACGGACTGCACACCCCGT	-22195	GGCTGGATTCTGTTTCTGTATTAAG
Allc	GCAGCTGATTGATTTGGCCTCTGAG	-19639	gggaagtatttttagaattatcctc
Cabs1	AACTATTACCTCGGAAGGAGACCAC	-5	TTCAGTAAATGACTGCACACCGGAT
Fam229b	TTGGCCTATTTTGGTAAGTCTATGT	-3266 +17*	TTCAGTAAATGACTGCACACCGGAT
Fscb	AATGTGAAGAACCTGAAGAGCCTAT	-3166	AGGTGTTAACTTAAACTAATAATTT
	AATGTGAAGAACCTGAAGAGCCTAT	-3164	TCAGGTGTTAACTTAAACTAATAAT
Hdgfl1	aggaagcgttgccagcatcctcgag	-1095	GGGACAGGCCAGTTGACATCAACTT
Iqca	CTAGGGGGAGTTCCCCCAACTTCGG	-107633	AGAGGAGGAAGAGAGTTTTAAGgta
Lelp1	aggcccctaagaacacctgcttact	-577bp+118#	AGTGAGGCACCATGGGCAACACTCC
Spata24	CCTTGCGAACAGAGGAAGTCCCCGC	-5638	ACTGTCTGTCTGCCTCCTATAGCAA
Tmem97	GGGCCCTGGCAGCTAGGCGCTGTGT	-8286	CGCTACCACAGCACTGCTAAAAAGG
Tmem114	TGGGCCGGAGCACGTGCGGGTGGGA	-15261	CCCATCTCAGTGGATGGCAACCTTT
Eddm3b	CCTGCTCTGATGTCCTCTCTAAAAG	-5	GCCCTGCTTCTGCTCCTGCTGTGCC
Lrcol1	GGTCCAACGTGAGATGTCTGTCTCG	-1196	AATGTGTGCCCTGGAGGAAGGTGAC

*+17: TTTCTTGAGCCCGCTAG

#+118:GAAGCTTTCAATGTGTTCAGTCTCTGGACCATCTCATTAAGAGCTGTCCTTGTCTGTGGACTTGGGGTATGTCTAGATGCCTGCACTCTGATTTTCTCTTACTTTGTTTCCATTCTAG

Supplementary Table S4. Testis weight relative to body weight in wild-type and mutant males.

Gene symbol	Testis/body weight ($\times 10^3$)	No. of males	No. of testis
Wild-type	3.0 ± 0.4	6	12
Fam229b	3.3 ± 0.1	3	6
Lqca	3.2 ± 0.0	2	4
Lelp1	4.2 ± 0.4	2	4
Spata24	3.8 ± 0.6	2	4
Tmem114	3.2 ± 0.5	3	6

Supplementary Table S5. The 13 knockout mouse lines in this study. The RBRC No. and CARD ID are available for the mouse lines that have been deposited as frozen sperm to Riken BioResource Research Center and Center for Animal Resources and Development at Kumamoto University, respectively.

Gene symbol	Gene name	RBRC No.	CARD ID
4921507P07Rik	RIKEN cDNA 4921507P07 gene	11050	2957
Allc	Allantoicase	11054	2961
Cabs1	Calcium binding protein, spermatid associated 1	10333	2703
Fam229b	Family with sequence similarity 229, member B	11035	2942
Fscb	Fibrous sheath CABYR binding protein	11063	2970
Hdgfl1	HDGF like 1	11018	2925
Iqca	IQ motif containing with AAA domain	TBD	TBD
Lelp1	Late cornified envelope-like proline-rich 1	TBD	TBD
Spata24	Spermatogenesis associated 24	TBD	TBD
Tmem97	Transmembrane protein 97	11025	2932
Eddm3b	Epididymal protein 3B	10345	2715
Lrcol1	Leucine rich colipase like 1	TBD	TBD
Tmem114	Transmembrane protein 114	TBD	TBD

Supplementary Table S6. Additional information about the 13 mouse genes analyzed in this study. The presence of transmembrane (TM) domains and a signal peptide was predicted using TMHMM and SignalP, respectively.

Gene symbol	NCBI UID	MGI ID	Chromosome	No. of TM domain	Signal peptide	Human orthologs
4921507P07Rik	70821	1918071	6	0	N	*C7orf31*
Allc	94041	2136971	6	0	N	*ALLC*
Cabs1	70977	1918227	5	0	N	*CABS1*
Fam229b	66337	1913587	10	0	N	*FAM229B*
Fscb	623046	3646964	12	0	N	*FSCB*
Hdgfl1	15192	1194493	13	0	N	*HDGFL1*
Iqca	74918	1922168	1	0	N	*IQCA1*
Lelp1	149018	1916582	1	0	N	*LELP1*
Spata24	71242	1918492	18	0	N	*SPATA24*
Tmem97	69071	1916321	10	4	N	*TMEM97*
Eddm3b	219026	2684921	14	0	Y	*EDDM3B*
Lrcol1	381667	2686525	5	1	Y	*LROCL1*
Tmem114	74720	1921970	16	4	Y	*TMEM114*

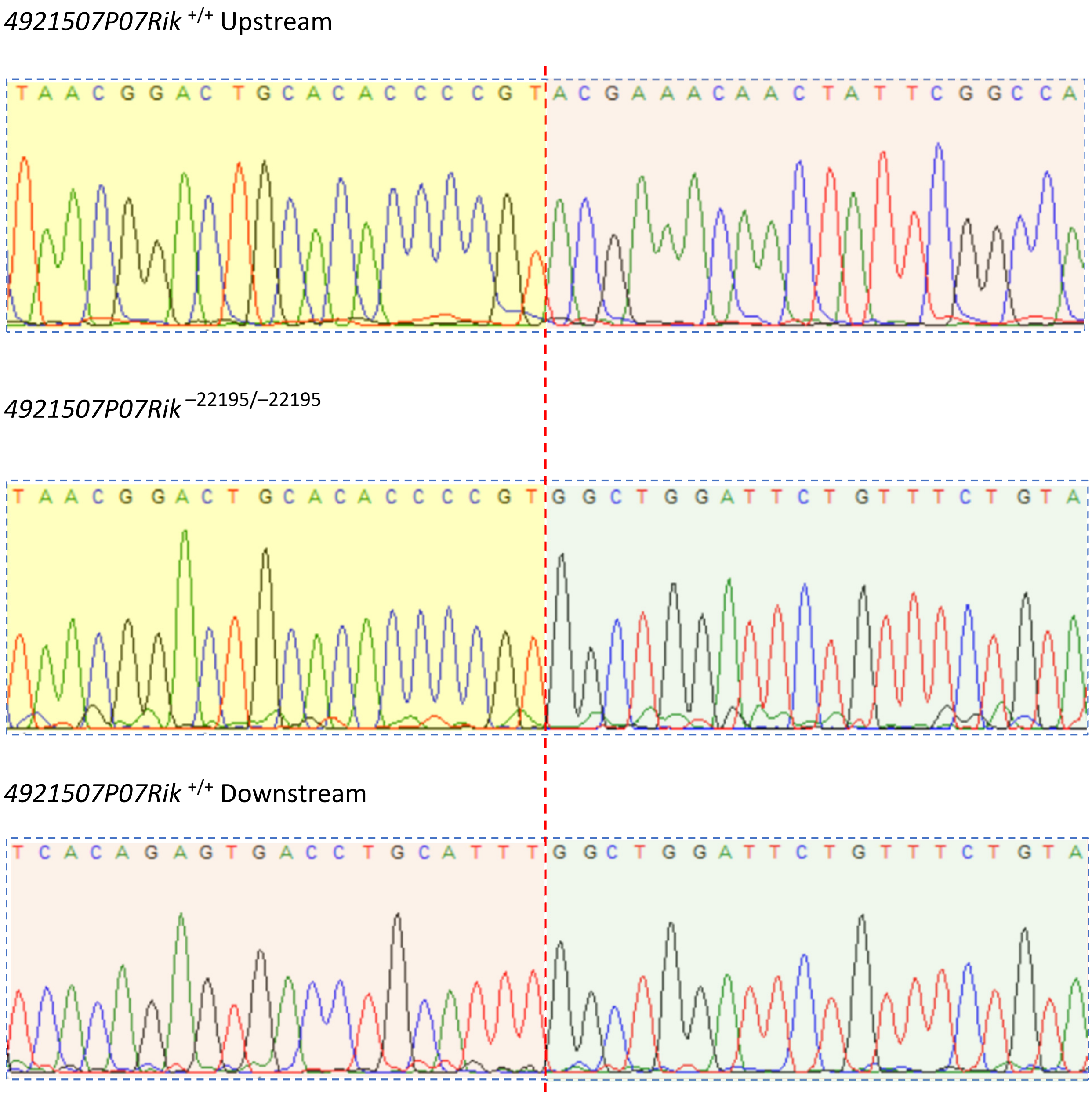

Supplementary Figure S1. Sanger sequencing of *4921507P07Rik* wild-type and knockout alleles. The upper and lower graphs indicate the upstream and downstream sequences of the wild-type allele, whereas the middle graph shows the knockout allele. The sequences deleted in the knockout mice are highlighted in red. The regions immediately upstream and downstream of the deletion are highlighted in yellow and green, respectively.

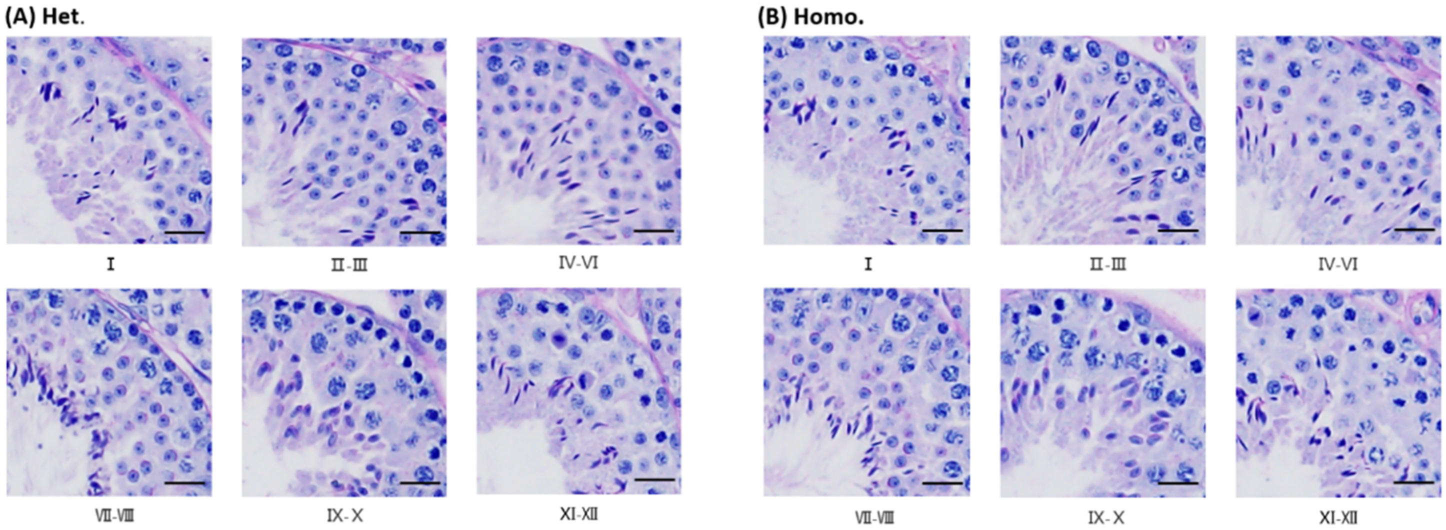

Supplementary Figure S2. Detailed analyses of spermatogenesis in **(A)** *Allc* heterozygous and **(B)** homozygous knockout mice. Scale bars = 20 µm.

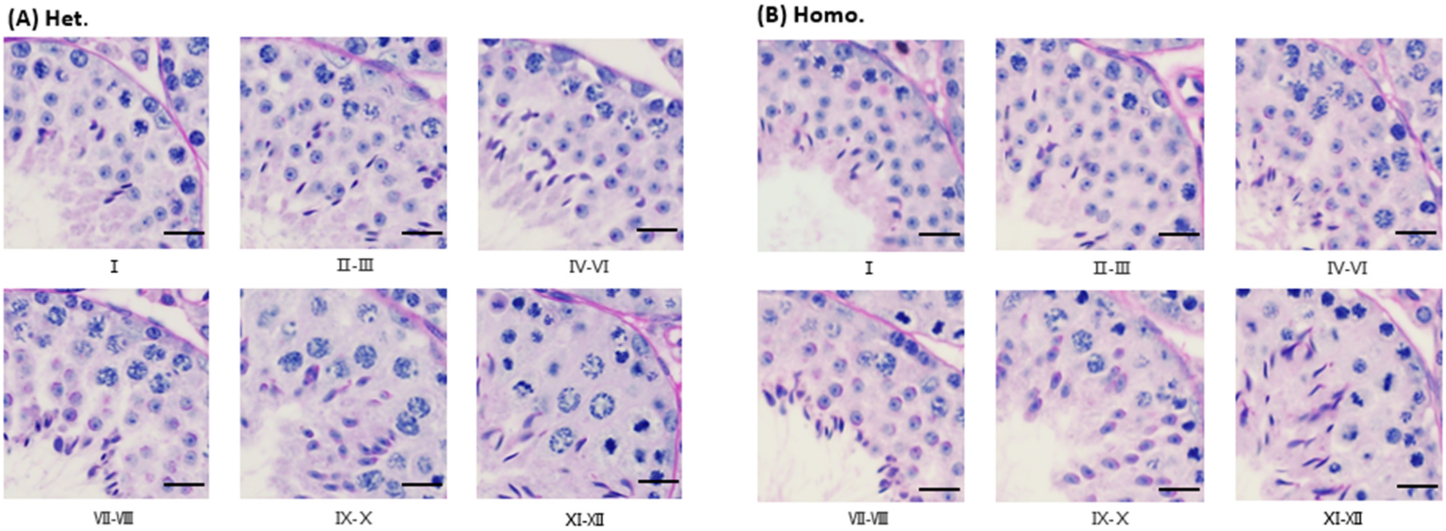

Supplementary Figure S3. Detailed analyses of spermatogenesis in **(A)** *Fscb* heterozygous and **(B)** homozygous knockout mice. Scale bars = 20 µm.

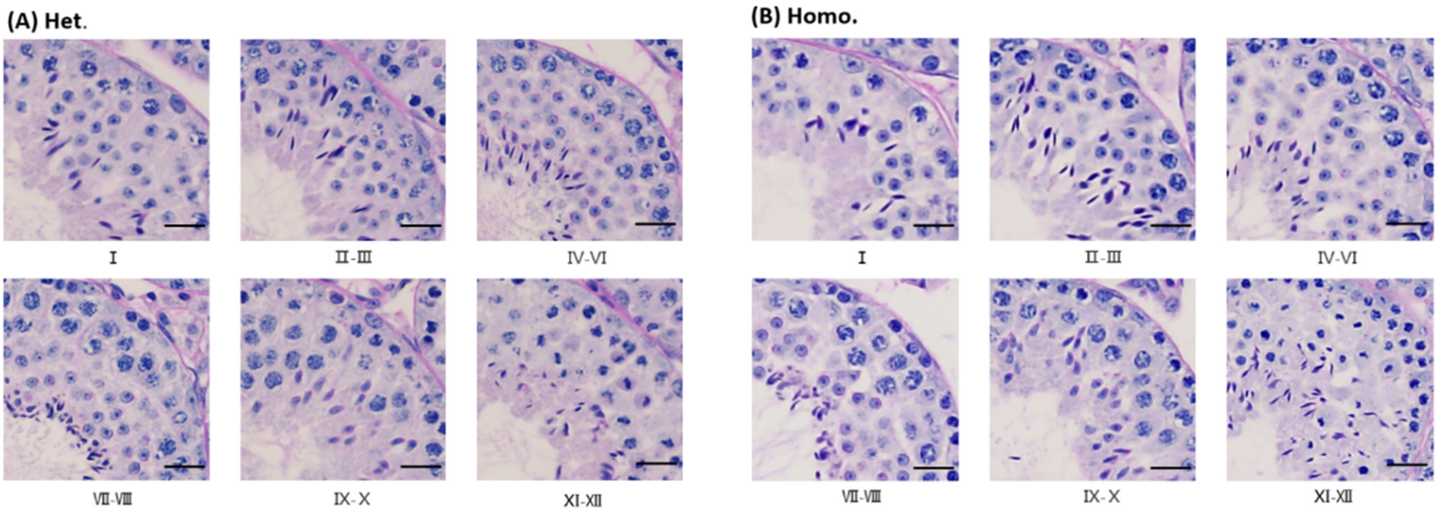

Supplementary Figure S4. Detailed analyses of spermatogenesis in **(A)** *4921507P07Rik* heterozygous and **(B)** homozygous knockout mice. Scale bars = 20 µm.

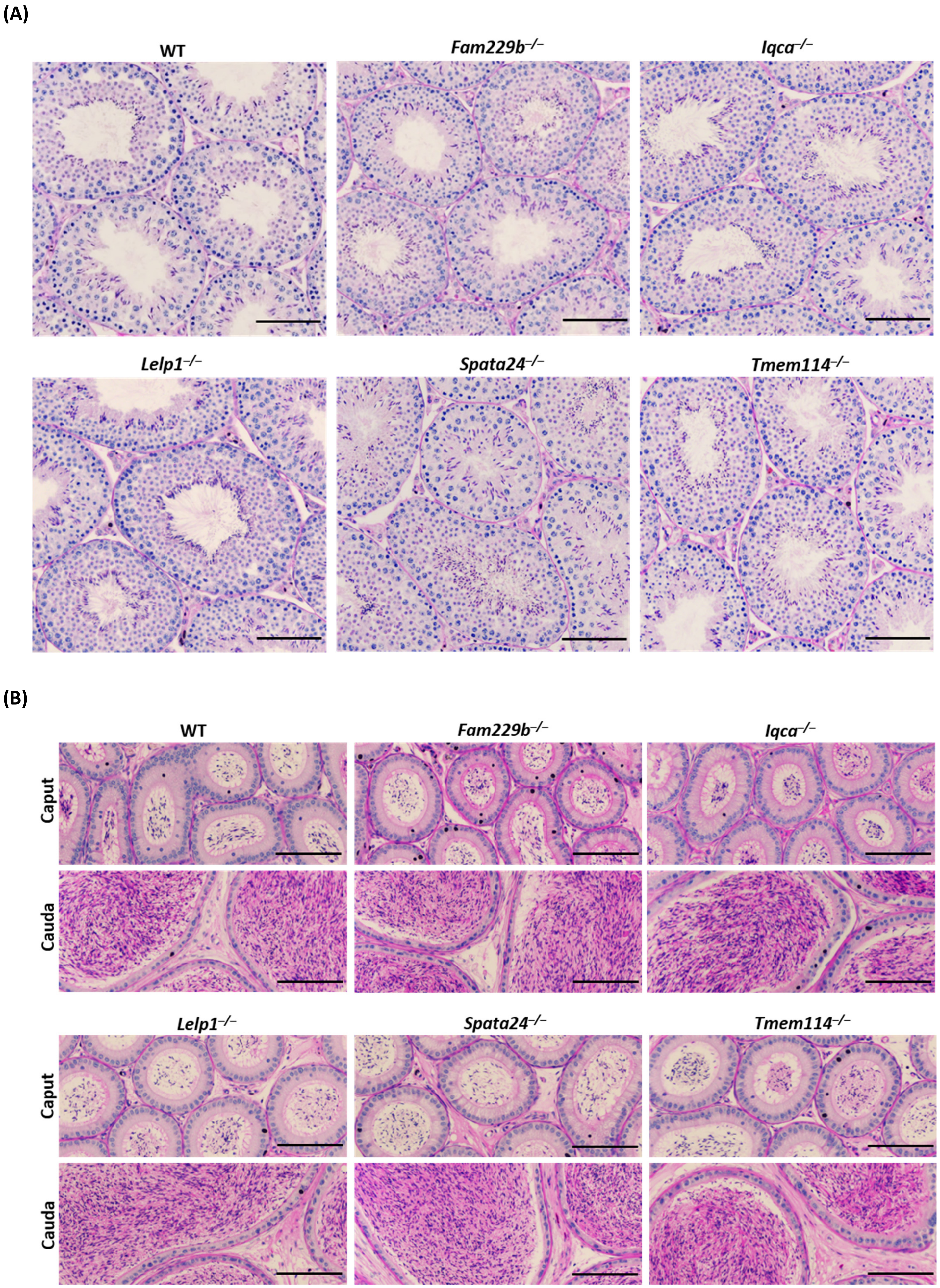

Supplementary Figure S5. Histological analyses of **(A)** testes and **(B)** epididymides in *Fam229b*, *Iqca*, *Lelp1*, *Spata24* and *Tmem114* knockout males. Scale bars = 100 µm.

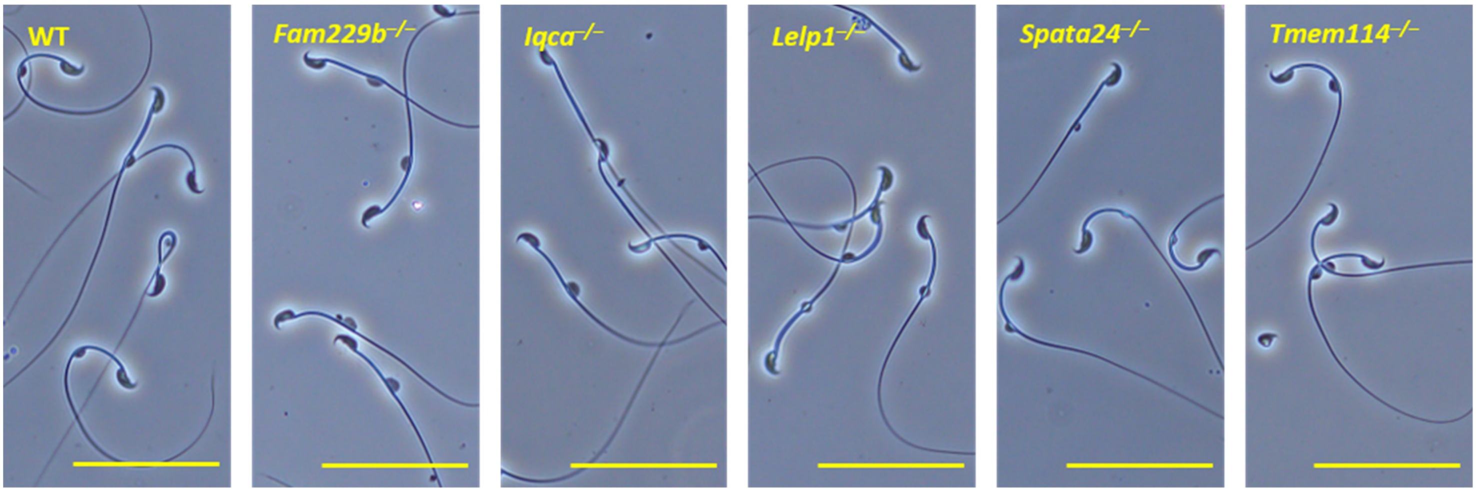

Supplementary Figure S6. Morphology of spermatozoa in *Fam229b*, *Iqac*, *Lelp1*, *Spata24* and *Tmem114* knockout males. Scale bars = 50 µm.

(A) 4921507P07Rik

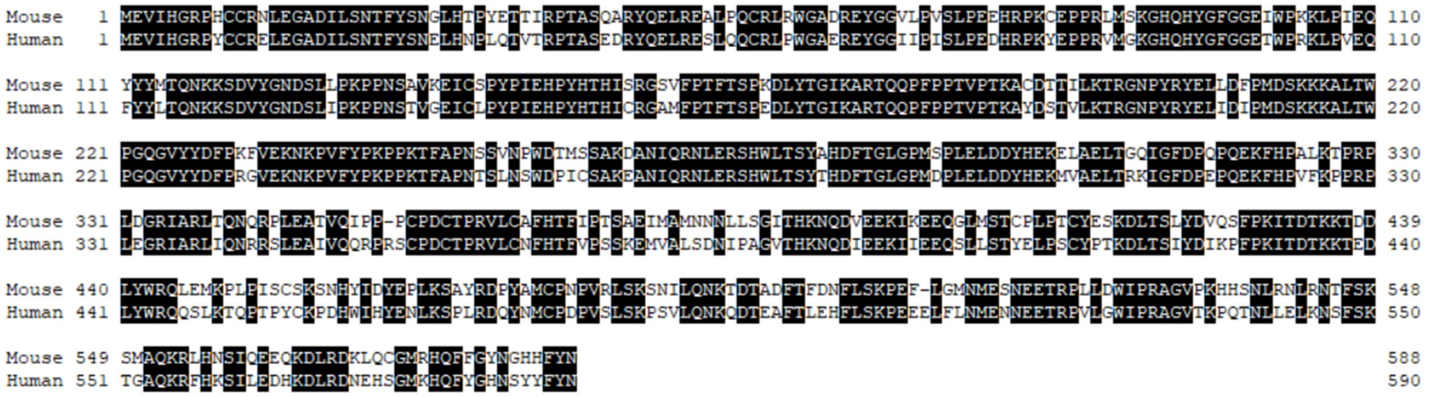

(B) ALLC

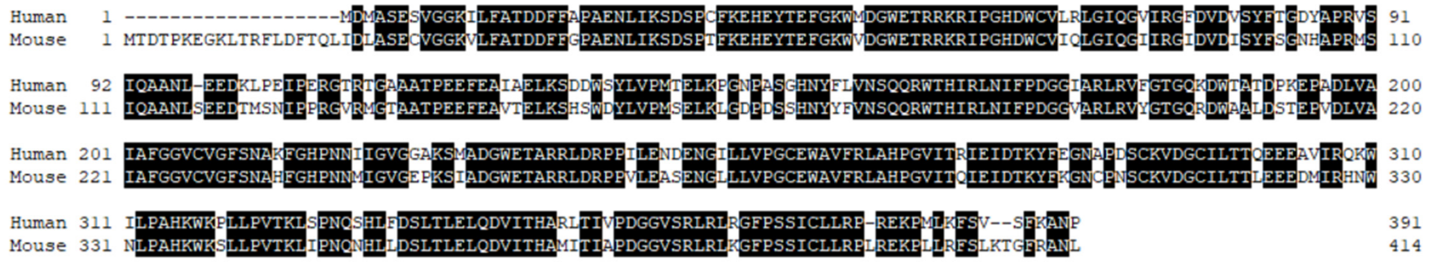

(C) CABS1

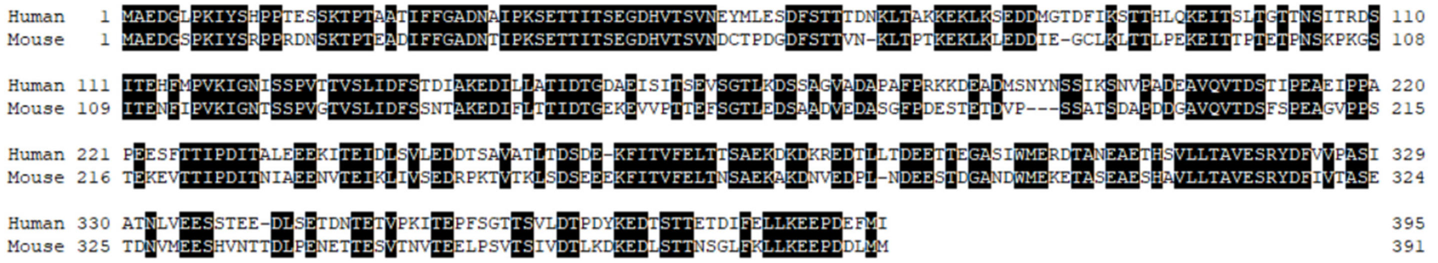

(D) FAM229B

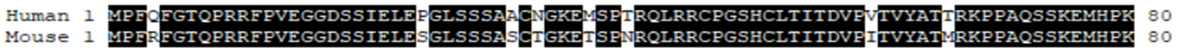

(E) FSCB

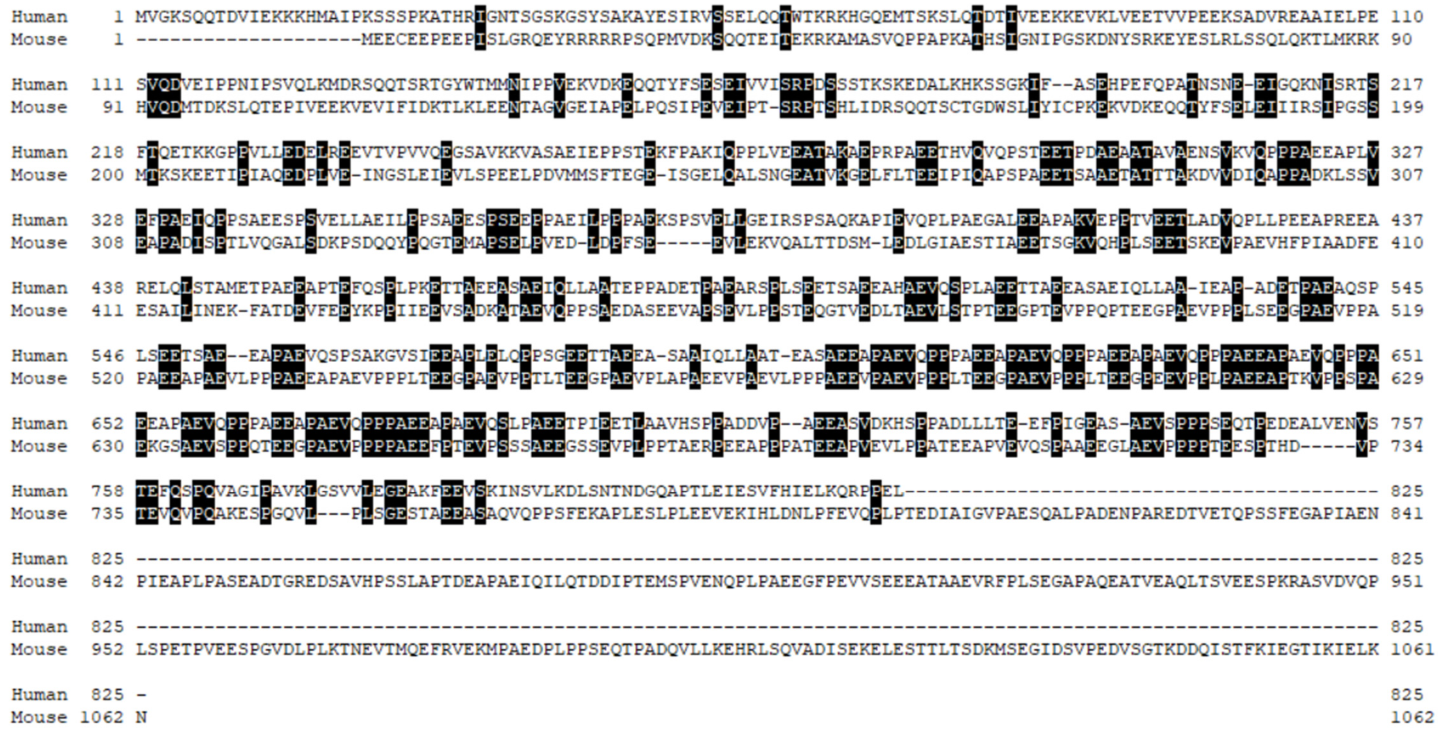

(F) HDGFL1

```
Human   1 MSAYGMPMYKSGDLVFAKLKGYAHWPARIEHMTQPNRYQVFFFGTHETAFLSPKRLFPYKECKEKFGKPNKRRGFSAGLWEIENNPTVQASD--CPLASEKGSGDGPWPE 108
Mouse   1 MSCFSRSKYKTGDLVFAKLKGYAHWPARIEHVAEANRYQVFFFGTHETALLGPRHLFPYEESKEKFGKPNKRRGFSEGLWEIEHDPMVEASSSLCS-EEDQSYTEDPGLA 109

Human 109 PEAAEGDEDKPTHAGGGGDELGKPDDDKPTEEEKGPLKRSAGD--PPEDAPKRPKEAAPDQEEEAEAERAAEAERAAAAAAATAVDEESPFLVAVENGSAPSEPGLVCEP 216
Mouse 110 EEPELGQELVQELEPEFMPELEAEPEMPETECEQEPEPQPAYDLLDAVEEPGLTKAEPGDQQAEHVQEKHPEVEAEAEAEAEAEAEAEAKAEVEEPGSLKRSAEDEEP 219

Human 217 PQPEEEELREEEVA-DEEASQEWHAEAPGGGDRDSL----------------------------                                            251
Mouse 220 HCPLKRPREAAPGALEMEPAEEREAEACPFVEEPDQAQEQLPPLEEEATEKAVQGLIVGEIEGL                                            283
```

(G) IQCA

```
Human   1 MSNAMYNKMWHQTQEALGALLDKEPQKMIEPQRNQVFIFQTLATFYVKYVQIFRNLENVYDQFVHPQKRILIRKVLDGVMGRILELKNEMVELELTEFHYFDDILQDLKL 110
Mouse   1 MSNLMYNKMWHQTQEALNSLLDKEFQKMSEPQANKVLVFQMLATFYINYVQIFRNMENVYDQIVHPQKRMLIRKVLDGVMGRILELKNEMVELEMTEFHYFDDILQDLKL 110

Human 111 APQQLDIPIPKYFLKEKLEVIKGREKILAQILADSGIDTSDMKYPVKSIPFDEAVKLIQIAERARQGRLRALFMKQIYLQEYRAKQSKMLGKKVTDTWAAALRIQKVWRR 220
Mouse 111 SPQQLDVPIPRYFLKERLEVIKGREKILARILTECGLNLP-VKYAAKSIALEEAVKLIQIAERARQGRLRALFMKQIFLQECRAKEMKLLGHRLLDTKLAALQIQKVWRG 219

Human 221 FHQRKETEKLREEEMIFLGMNPPPLFNEVSATVIQAEKVDRLRNEVQIKHEEDYREALVTIKNDLKLIEGVDIKENLQDQIRHWFIECRNLTGTFPDYPDVEEGGSAIIF 330
Mouse 220 FHQCKKTVKEREEEMVFLGMKPSPLFNEVSDAIVQSKQVTNLRDEVQLKHEQDYQEALVNIKEDLKMLEGPDIKEHLQDQIRQWFIECRNLTGTFPDYPNEEDGGSALIF 329

Human 331 SDKTIQQE-----------------------------------------EDEKWKMSPSLFLPAMKEGCNAYKEIWMKKDESWNFSQDYDPELIKEEKRKELQSEIRIQ 398
Mouse 330 SNKTPEQVMGDIIATQEEEEKLKKKKKKEDKENKGKKGKKEKKEKKEKKDEDEEWKMSPSLFLQLMEEGNSLYKDIWLNKDESWNFPQDYDPELIKEEKRKELEMEIRVQ 439

Human 399 VDELMRQELKNLKLAVDREREREPVKAGK-KKDKKGKKGKKKEKKAKKDKDLTADRTIESLYKELVEEGLLIQALKVNLSDYIGEYSYLGTTLRQVSIEPMPSLLDVRQLI 507
Mouse 440 VDELMRQELKNLKLAVNREMEIPPRQKKGKKKNKGRKGRR-GKRGKKEKDLTADRTIESLYQELVEKGLLIQALKVNLSDYIGEYSYLGTTLRQVAIEPMPSLLDVRQLI 548

Human 508 TLYGIWPLGSAAVHEKAPLVKSLLLAGPSGVGKKMLVHAICTETGANLFNLSSSNIAGKYPGKNGLQMMLHAVFKVARQLQPSVVWIEDTEKTFYKKVPNAEKMNEPKRL 617
Mouse 549 TLYGILPLGSAEVHERAPLVKSLLLAGPSGVGKKMLVHAICTETGANLFNLSALNIAGKYPGKTGLQMMIHLVFKVARQLQPSVVWIQDTEKTFYKKVPQAEKRFEPKRL 658

Human 618 KKHLPQILKLLKPDDRILIVGTTRRPFDAELQSFCKVYQKIILVPRPDYASRYVLWKQIIERNGGVLTSALNVSCLAKVTDGFTQGHIVEVVKGVLTDQRIRRQIHKPLT 727
Mouse 659 KRYLPKLLKLLKPDDRILIVGTTHRPFDAELQPFCRVYQKIILVPRPDYASRYVLWKEIILRNGGKLTNSLNISCLSKVTDGFTQGQIVQVIKDVLTERRLRQQAHKPLT 768

Human 728 AVEFITAITSMNPVYKEEEESFKNWYAKTPLGKKRALAITGGSTEKAKDKGKRK-------                                                 781
Mouse 769 AIEFITMMTNMNPVYREEEESFKNWYAKTPLGKKRVLSLTVGNKEKEKDKGKKGKRGKKKK                                                 829
```

(H) LELP1

```
Human   1 MSSDDKSKSNDPKTEPKNCDPKCEQKCESKCQPSCLKKLLQRCFEKCPWEKCPAPPKCLPCPSQSPSS--CPPQPCTKPCPPKCPSSCPHACPPP--CPPPE-------- 98
Mouse   1 ---MSSDKSSDSKNGLKNCDPRCEQKCETKCQPSCLNKLLQRCSEKCSLDKCPPSPNCPPCPPCPPCPLVCQSQ-CSTPTSPLCPPLCSPRCSGTLACCPPSCPQKGCVK 106

Human  98 --------------                                                                                               98
Mouse 107 PCPPKCPSPCLPRK                                                                                               120
```

(I) SPATA24

```
Human   1 MATPLGWSKAGSGSVCLALDQLRDVIESQEELIHQLRNVMVLQDENFVSKEEFQAVEKKLVEEKAAHAKTKVLLAKEEEKLQFALGEVEVLSKQLEKEKLAFEKALSSVK 110
Mouse   1 MATPLGWSQGGSGSVCLAFDQLRDVIESQEELIHQLRNVMVLQDENFVSKEEFHEIEKKLVEEKAAHAKTKALLAKEEEKLQFALGEVEVLSKQLEKEKMAFEKALSSVK 110

Human 111 SKVLQESSKKDQLITKCNEIESHIIKQEDILNGKENEIKELQQVISQQKQIFRNHMSDFRIQKQQESYMAQVLDQKHKKASGTRQARSHQHPREK               205
Mouse 111 SRVLQESSKKDQLITKCNEIESHIIKQEDILNGKENEIKELQQVISQQKQNFRNHISDFRIQKQQETYMAQVLDQKRKKATGMRRARSRQCSREK               205
```

(J) TMEM97

```
Human   1 MGAPATRRCVEWLLGLYFLSHIPITLFMDLQAVLPRELYPVEFRNLLKWYAKEFKDPLLQEPPAWFKSFLFCELVFQLPFFPIATYAFLKGSCKWIRTPAIIYSVHTMTT 110
Mouse   1 MGALAARRCVEWLLGLYFVSHIPITLFIDLQAVLPPELYPQEFSNLLRWYSKEFKDPLMQEPPVWFKSFLLCELVFQLPFFPIAAYAFFKGSCRWIRIPAIIYAAHTITT 110

Human 111 LIPILSTFLFEDFSKASGFKGQRPETLHERLTLVSVYAPYLLIPFILLIFMLRSPYYKYEEKRKKK                                            176
Mouse 111 LIPILYTLLFEDFSKAVAFKGQRPESFRERLTLVGVYAPYLIIPLILLLFMLRNPYYKYEEKRKKK                                            176
```

(K) EDDM3B

```
Human   1 ------MASSLKIWGTLLALLCILCTLLVQSKEVSWREFMKQHYLSPSREFREYKCDVLMRENEALKDKSSHMFIYISWYKIEHICTSDNWMDRFRNAYVWVQNPLKVLK 104
Mouse   1 MSLVPALMSSLKAWALLLLLLCLQHSLLAQST--SRRQFMEHHHLSPNKMFSDYNCDVLMTDK-AVKPKLSHMFVYMTWYKVEHICIGSNWRDRYKNMYIWAQTPIKVLR 107

Human 105 CHQENSKNSYTESRSFNYIEFHCSMDGYVDSIEDLKMVEPIGN                                                                   147
Mouse 108 CQWESLKNRYTERRSYSYVQFHCNADGYVDSIEDIKALEPIL-                                                                   149
```

(L) LRCOL1

```
Human   1 MAGPGWTLLLLLLLLLLLGSMAGYGPQKKLNLSHKGIGEPCRRHEECQSNCCTINSLAPHTLCTPKTIFLQCLPWRKPNGYRCSHDSECQSSCCVRNNS-PQELCTPQSV 109
Mouse   1 MSVSVWPPLLLLLLLLLLWAVPTFQDKNTRVSAYKGIGEMCRNNSECQSDCCVTNSLNPQKFCTSQTVFLECVPWRKPNGFLCEENTECHSNCCIRTSSNPDRFCSSKTI 110

Human 110 FLQCVPWRKPNGDFCSSHQECHSQCCIQLREYSPF-RCIPRTGILAQCLPL                                                           159
Mouse 111 FMQCISWRKPEGAICQHHLECWDLCCLPLSENSPSSHCTKRTGLLALCLPV                                                           161
```

(M) TMEM114

```
Human   1 MRVHLGGLAGAAALTGALSFVLLAAAIGTDFWYIIDTERLERTGPGAQDLLGSINRSQPEPLSSHSGLWRTCRVQSPCTPLMNPFRLENVTVSESSRQLLTMHGTFVILL 110
Mouse   1 MRVRLGALAGAAALSGALSFVLLAAAIGTDFWYIIDTERLERSSQRMRDQ-GPANRSQQEPLSSHSGLWRTCRVQSSCTPLMNPFWQENVTVSDSSRQLLTMHGTFVILL 109

Human 111 PLSLILMVFGGMTGFLSFLLQAYLLLLLTGILFLFGAMVTLAGISVYIAYSAAAFREALCLLEEKALLDQVDISFGWSLALGWISFIAELLTGAAFLAAARELSLRRRQD 220
Mouse 110 PLSLIVMVFGGMTGFLSFLLRAHLLLLLTGILFLFGAMVTLTGISIYIAYSAVAFREAVCLLEERALLDQVDIRFGWSLALGWISFVSELLTGVVFLAAARALSLSQRQD 219

Human 221 QAI                                                                                                            223
Mouse 220 QAI                                                                                                            222
```

Supplementary Figure S7. Alignment of mouse and human peptide sequences encoded by each gene. Dark regions indicate identities between mouse and human sequences. **(A)** 4921507P07Rik. **(B)** ALLC. **(C)** CABS1 **(D)** FAM229B. **(E)** FSCB. **(F)** HDGFL1. **(G)** IQCA. **(H)** LELP1. **(I)** SPATA24. **(J)** TMEM97. **(K)** EDDM3B. **(L)** LRCOL1. **(M)** TMEM114.

Cabs1

```
Wild-type   MAEDGSPKIYSRPPRDNSKTPTEADIFFGADNTIPKSETTITSEGDHVTS...LKEEPDDLMM*
Mutant      MAEDGSPKIYSRPPRDNSKTPTEADIFFGADNTIPKSETTITSEGDHFSK*
```

Supplementary Figure S8. Amino acid sequences encoded by wild-type and mutant *Cabs1*.

Eddm3b

```
Wild-type   MSLVPALMSSLKAWALLLLLLLCLQ...EDIKALEPIL*
Mutant      MSLVPALMSSLKGPASAPAVPAA*
```

Supplementary Figure S9. Amino acid sequences encoded by wild-type and mutant *Eddm3b*.
